# The dangers of castor oil tea in neonates in rural Haiti: A case report

**DOI:** 10.3389/fped.2023.877550

**Published:** 2023-03-06

**Authors:** Wilhermine Jean Baptiste, Michelucia Casseus, Alka Dev, Patrice Joseph, Peter F. Wright

**Affiliations:** ^1^ Department of Pediatrics, Dartmouth Hitchcock Medical Center (DHMC), Lebanon, NH, United States; ^2^ Department of Pediatrics, Hopital Immacule Conception (HIC), Les Cayes, Haiti; ^3^ Department of Medicine, Gheskio Centers, Port-au-Prince, Haiti

**Keywords:** neonatal, sepsis, castor oil, ricin, Haiti, toxin

## Abstract

During a 2-year period, eight cases of a distinct illness were seen among 1,424 neonates admitted to a newly established neonatal care unit in southern Haiti. The newborns presented with a picture of sepsis with shock, vomiting, hypotonia, lethargy, and abdominal distention. Five cases proved fatal and another case left the hospital against advice in extremis with little chance of survival. In each case, the illness was associated with a history of ingestion of teas that included castor oil, known as lok in Haitian Creole. The presumptive cause of the illness was established by the presence of a dark, oily substance in drainage from the nares and nasogastric tubes and by subsequent admission on direct questioning of the caregivers, who said that the infants had been given large amounts of lok. The castor oil tea had been given to three infants in the immediate neonatal period where its use is attributed to encouraging the passage of meconium. The five remaining infants were between 15 and 30 days of life when they were given lok shortly before admission to the neonatal unit for treatment of an undefined illness. All of them were term infants with no identified risks at birth. As nasogastric tubes are not routinely placed in sick neonates, and the parents did not volunteer information about lok administration, the practice may be more widespread than that recorded here. Although our data are confined to observations in Haiti, the use of traditional medicines is a globally widespread phenomenon. Attention must be drawn to the potential toxicity of such preparations and means found to ban their use in neonates.

## Introduction

Haiti is one of the poorest countries in the Americas. It has widespread beliefs in traditional as well as allopathic medicine, especially with regard to maternal and child health (1–4). Traditional practices and beliefs play an important role in healthcare delivery. A traditional birth attendant with no formal training living in the community is often considered to be the most trusted adviser for a pregnant woman (5). In rural areas, 60% of women turn to traditional birth attendants for deliveries in their homes (6). However, much of their advice has no basis in established medical practice and includes avoidance of colostrum and the administration of castor oil lok to clear meconium from the gastrointestinal tract (7). The castor oil, also called maskreti oil or the oil of Palma Christi, is usually prepared locally.

Teams from Groupe Haitien d’Etudes du Sarcome de Kaposi et des Infections Opportunistes (GHESKIO), a Haitian research, training, and treatment center in Port-au-Prince, and the Dartmouth-Hitchcock Medical Center worked with the staff and leadership of Hopital Immaculee Conception (HIC) in Les Cayes, a city on the southern coast of Haiti, to establish a neonatal unit in that hospital. HIC serves as the referral facility for the Department of the South, one of the two facilities equipped to provide tertiary obstetrical and neonatal care for the region.

There are 3,000–4,000 routine and complicated annual deliveries performed at HIC (8).

With the initiation of the GHESKIO/Dartmouth project, new staff were recruited including two Haitian pediatricians with training in neonatology. A detailed electronic database of all admissions was kept, enabling the documentation of individual case reports and the overall impact of the program. Approval for maintenance, analysis, and publication of findings was obtained from the GHESKIO and Dartmouth IRBs. The Dartmouth IRB specifically reviewed this manuscript and agreed that it respected patient confidentiality and did not meet criteria for obtaining individual consent.

During the first 2 years of the ward's operation, there were 1,424 admissions to the ward including the eight patients with lok poisoning reported in this paper. Conversations with healthcare providers in Haiti indicated that although the use of lok was decreasing in urban areas, the practice remained common in rural areas. There are reports of the use of castor oil in Haitian newborns from the 1980s (9). While complications have been recognized, the use of lok has not been reported as a cause of serious illness or death among Haitian neonates (7).

## Case findings

The eight patients reported here came from different parts of the southern region of Haiti. There was no temporal or geographic clustering of these patient cases. The available information on these eight lok cases is presented in Table 1. The ages of the eight children suggested that there were two periods when an infant was at risk of being given lok, either in the immediate neonatal period or at 2–3 weeks of age. We could not assume that the parent was responsible for giving the tea, as other women in the family, such as grandmothers and traditional birth attendants, play a prominent role in Haitian newborn care.

**Table 1:** Cases of castor oil poisoning reported at HIC, Les Cayes, Haiti.

Place of birth	Age at presentation (days)	Source of *lok*	Duration of hospitalization (days)	Outcomes	Mother's age (years)
Home	26–30	Parent	9	Left against advice	21–25
HIC	<5	Parent	6	Died	11–15
Home	<5	Family member	1	Died	25–30
Port a Piment Hospital	21–25	Parent	2	Discharged	35–40
HIC	16–20	Parents	19	Died	31–35
HIC	<5	Parent	1	Died	31–35
HIC	16–20	Parent	1	Died	21–25
HIC	16–20	Parent	8	Discharged	21–25

HIC, Hopital Immaculee Conception.

A brief description of a representative patient is as follows:

A term baby was born to a young mother after 18 h of labor at HIC. It was her first child and the pregnancy was unplanned. She had no routine prenatal care beyond being vaccinated with tetanus during pregnancy and having negative screens for HIV and syphilis. She had a urinary tract infection that was untreated. There was no diabetes or hypertension. The baby was born with a cephalic presentation with a spontaneous cry and was discharged home without pediatric evaluation.

On the third day of life, lok was given by a family member at home. The baby presented to the nursery the following day appearing to be septic. The infant was febrile, dyspneic, and dehydrated with yellow secretions from the mouth. He had a poor suck. Bowel sounds were present and there was no abdominal distention. After use of a nasogastric tube, dark oily secretions were aspirated. Over the next day, the infant developed seizures, and in spite of anticonvulsants and antibiotic therapy, he died on the sixth day of hospitalization.

In some of the patients, the clinical picture looked as though it included an aspiration pneumonia with the bitterness of the teas and the distastefulness of castor potentially contributing to vomiting and aspiration.

## Discussion

Historically, castor oil has been used as a laxative, purgative, and vermicide. Its use was documented in Europe in the 1700s (10) and Appalachia as recently as the mid-20th century (11). A text from 1785 suggests that it may have been introduced to England from the Caribbean (12). Recent use in India is also documented (13). There is widespread use of castor oil for skin and hair care with no documented toxicity.

In reaching out to physicians who have worked across Haiti (see the Acknowledgements section), we learned that the administration of lok or castor oil tea is an ongoing practice throughout rural Haiti. Descriptions of the preparation of lok vary. The simpler preparations consist of castor oil, salt, and nutmeg. More complex mixtures include castor oil, nutmeg, garlic salt, and leaves.

Another description was that lok could include castor oil, nutmeg, cane syrup, garlic, water, and cockroaches, all put in a piece of tissue and boiled for a few minutes. Up to 161 local plants have been identified for use in Haitian traditional medicine (2). Thus, the composition of lok in Haiti invariably has castor oil but differs in other components from one region to another. The practice of using lok declined in Haiti following an information campaign orchestrated in the 1980s by the Ministry of Health on the harmful effects of this practice (14). However, we document that its use persists among some families in rural Haiti.

In Haiti, the production of castor oil remains marginal and artisanal. This poses a particular safety problem in that castor oil is extracted from castor beans whose shell contains a potent poison, ricin, which acts as a type 2 ribosome-inactivating protein (RIP) (15). Ricin lectin is inactivated by heat, which is used to roast or boil the castor beans with the recommendation that it be at 80 degrees C for one hour. The homemade preparation of this oil may not ensure the above conditions aimed at inactivating the ricin. We postulate the residual ricin could be one of the causes of the neonatal toxicity of lok. Ingestion of ricin causes pain, inflammation, and hemorrhage in the mucosal membranes of the gastrointestinal system (16). With ricin ingestion, gastrointestinal symptoms quickly progress to severe nausea, vomiting, diarrhea, and dysphagia. Hemorrhage causes melena and hematemesis. Hypovolemia caused by gastrointestinal fluid loss can lead to organ failure in the pancreas, kidney, liver, and gastrointestinal tract and progress to shock (16). An extensive review of human and animal toxicity from castor bean ingestion reports an illness consistent with that seen in the infants and documents the toxicity of even small doses of ricin (17). It is noted that this comprehensive review did not mention neonatal toxicity.

In addition, castor oil itself is a laxative. The ingredients of the tea, even if briefly heated, clearly are not sterile. Finally, in the patient cases in this study, the lok by itself may not be causative if the castor oil tea was given because an infant was already sick. We believe that the latter possibility is remote but should be considered. Initially, the parents were reluctant to volunteer information that they had given their infants lok.

## Limitations

The limitations of our observations need to be acknowledged. These limitations include the fact that we were not aware how the lok was made and given to the infants reported in this study nor did we have any samples of these lok preparations that could have been analyzed for ricin content. Blood cultures and laboratory documentation of hepatic and renal status could not be done. Radiology was very limited, and no autopsies were performed. However, the primary clinicians and other authors are confident that lok ingestion is associated with the outcomes reported, but additional documentation of the chemical nature of the gastric aspirates could be supportive of our clinical association.

## Conclusion

This article shows that the use of castor oil is an ongoing practice in newborn Haitian children. Castor oil teas are given in the belief that they help pass meconium stool or that they have therapeutic value for minor illnesses. Usually, these teas may be given in small doses, but on occasion, as with the patients in this study, large volumes may be given. The use of lok is an example of traditional medicine that is perpetuated by deeply embedded traditions. However, we have documented adverse outcomes that can accompany this practice. We believe that this practice is underdocumented and poses a risk to Haitian neonates in the first month of life. Its use in the developing world may not be limited to Haiti. It is a form of poisoning that can be seen in immigrants to the United States and other developed countries through the importation of herbal remedies.

The level of awareness of the harmful effects of certain traditional medicine practices, especially for vulnerable populations such as neonates, needs to be increased among medical practitioners, traditional birth attendants, and caretakers in Haiti. Of the eight cases of castor oil poisoning identified at HIC, five resulted in fatal outcomes. In an infant who returns to the hospital after discharge with nonspecific signs of abdominal distress and possible sepsis, the use of a nasogastric tube would confirm the presence of lok in the stomach. Continued reinforcement of the necessity of exclusively breastfeeding would help limit lok administration. The best way to make this happen is possibly by coordinating with the Ministry of Health and Haitian Pediatric Societies. Radio, text messages, or “teledjol,” the highly effective word-of-mouth system that exists in Haiti, are other ways of reaching people in the rural areas.

## Data Availability

The original contributions presented in the study are included in the article, further inquiries can be directed to the corresponding author.
